# Supplementary material for: MethNet: a robust approach to identify regulatory hubs and their distal targets in cancer
Source: Res Sq. 2023 Jul 31:rs.3.rs-3150386. Preprint. [Version 1] doi: 10.21203/rs.3.rs-3150386/v1 (PMC10418566; doi:10.21203/rs.3.rs-3150386/v1)
Supplement: Supplement 1 [file NIHPPRS3150386V1-supplement-1.pdf]

Meemental Figures

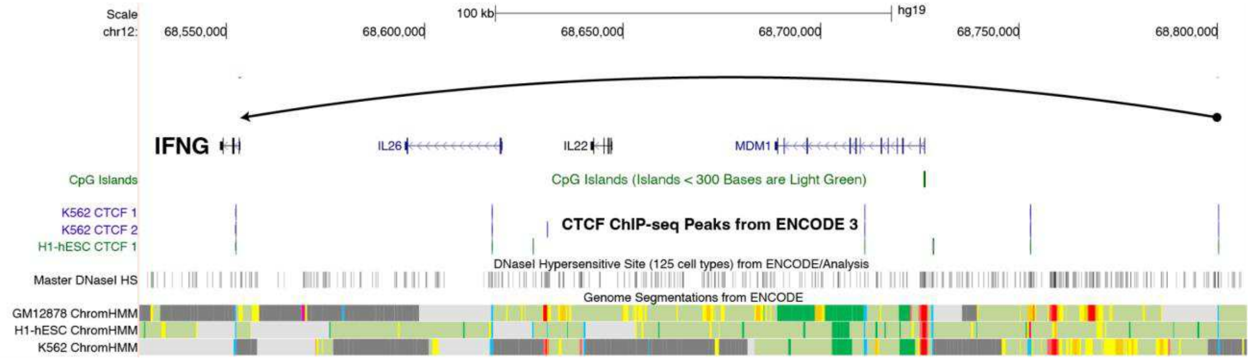

Figure S1: An example of a repressive MethNet association. IFNγ expression is regulated by a CTCF binding site located 250 kb upstream of its promoter. UCSC Genome Browser session highlighting the MethNet association on top track.

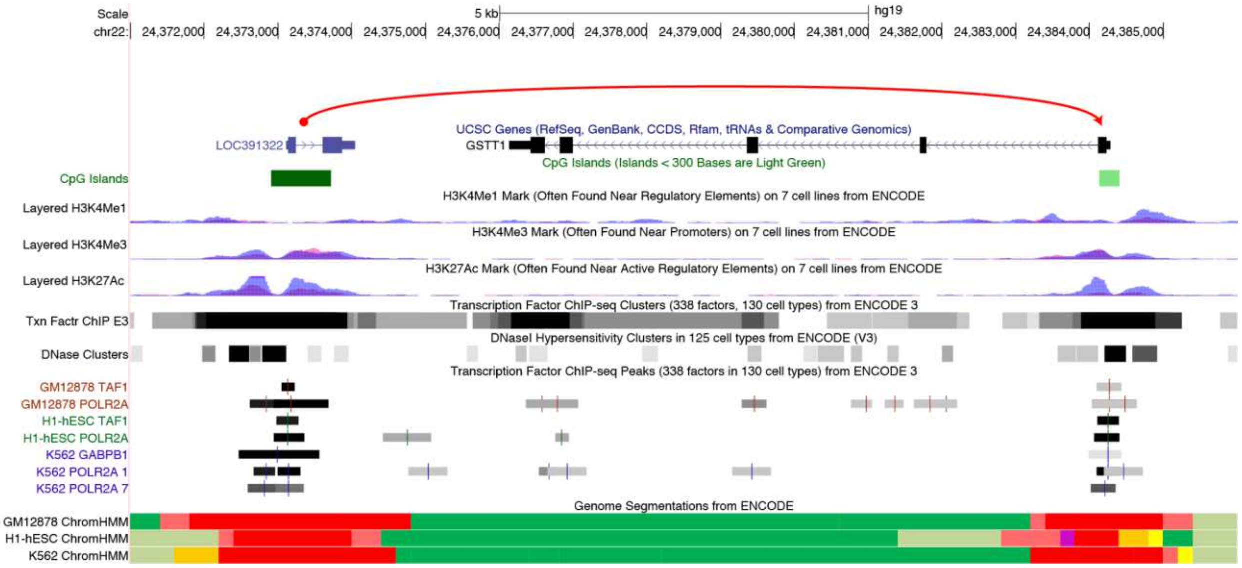

Figure S2: An example of an activating MethNet association. *GSTT1* is regulated by the promoter of a downstream non-coding gene. UCSC Genome Browser session highlighting the MethNet association on top track.

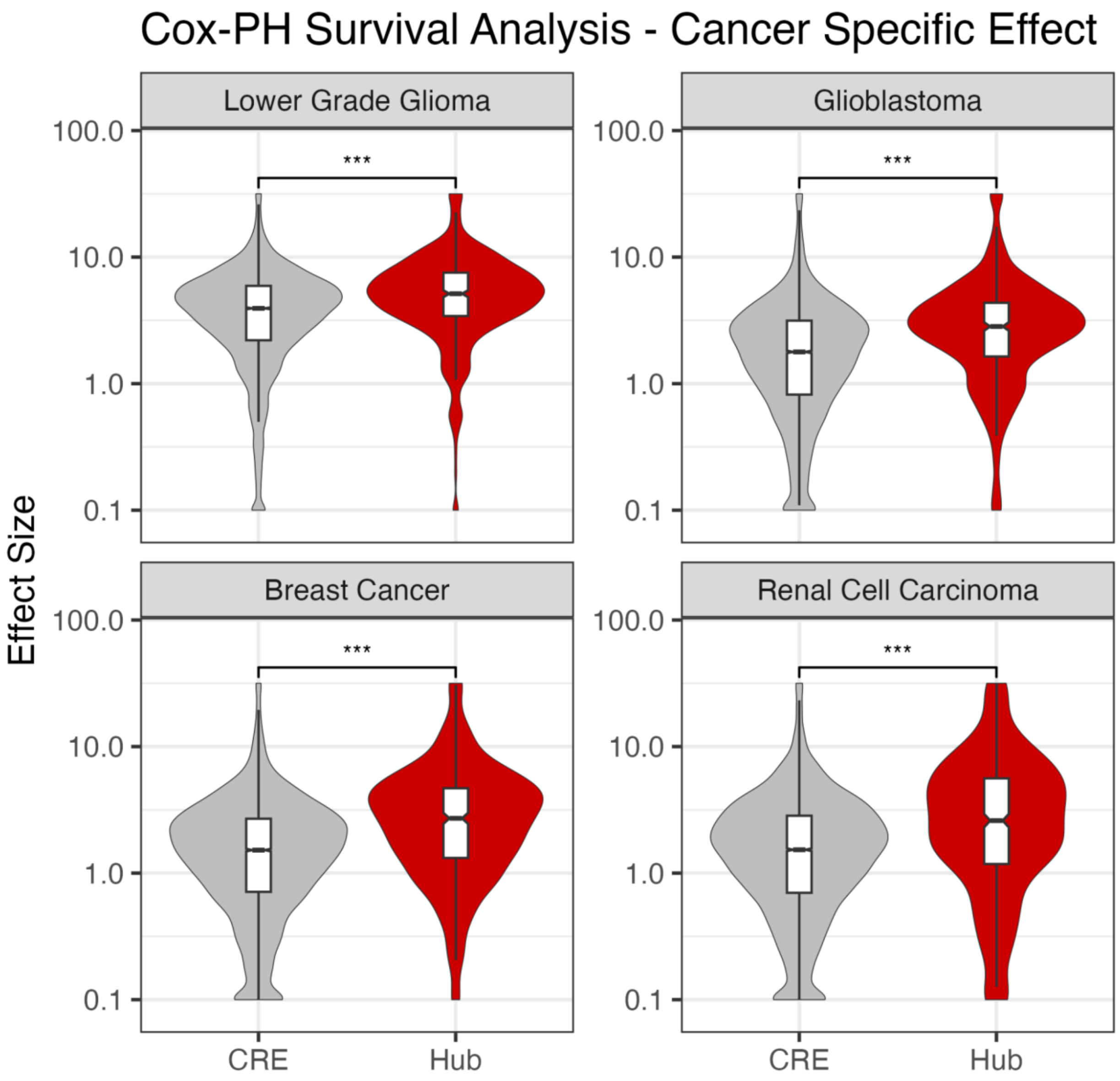

Figure S3: Cancer-specific effects of methylation of hubs vs non-hub elements. Distribution of the absolute marginal effect methylation of hub and non-hubs elements for TCGA datasets that were affect more than the rest.

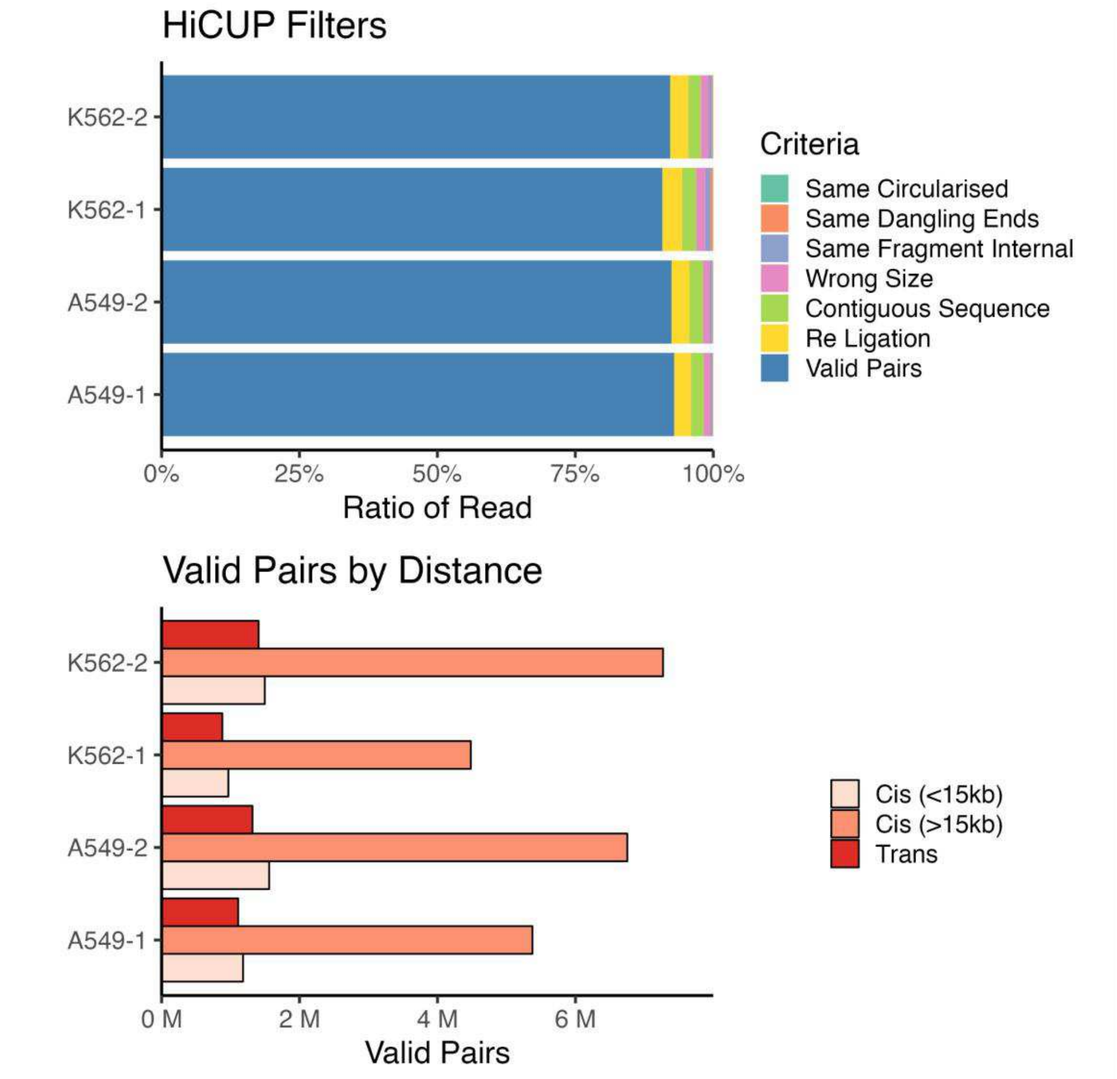

Figure S4: Quality control metrics of promoter capture Hi-C reads.

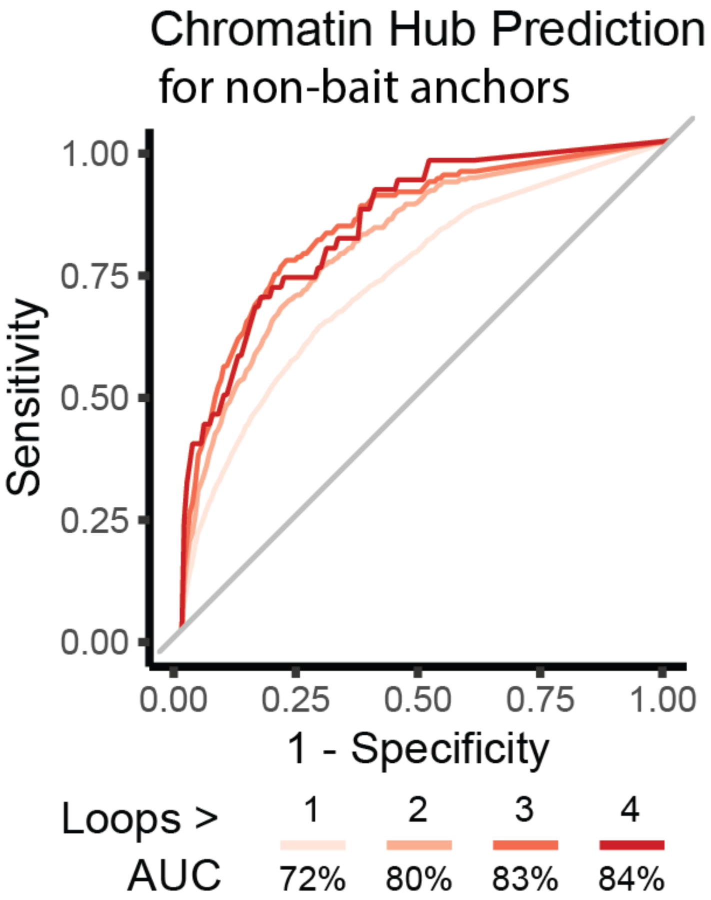

Figure S5: MethNet potential predictive power for chromatin hubs. We analyzed intergenic hubs separately from promoter hubs because the latter are enriched in our data because of the experimental design. As a result, we used thresholds that were more lenient.

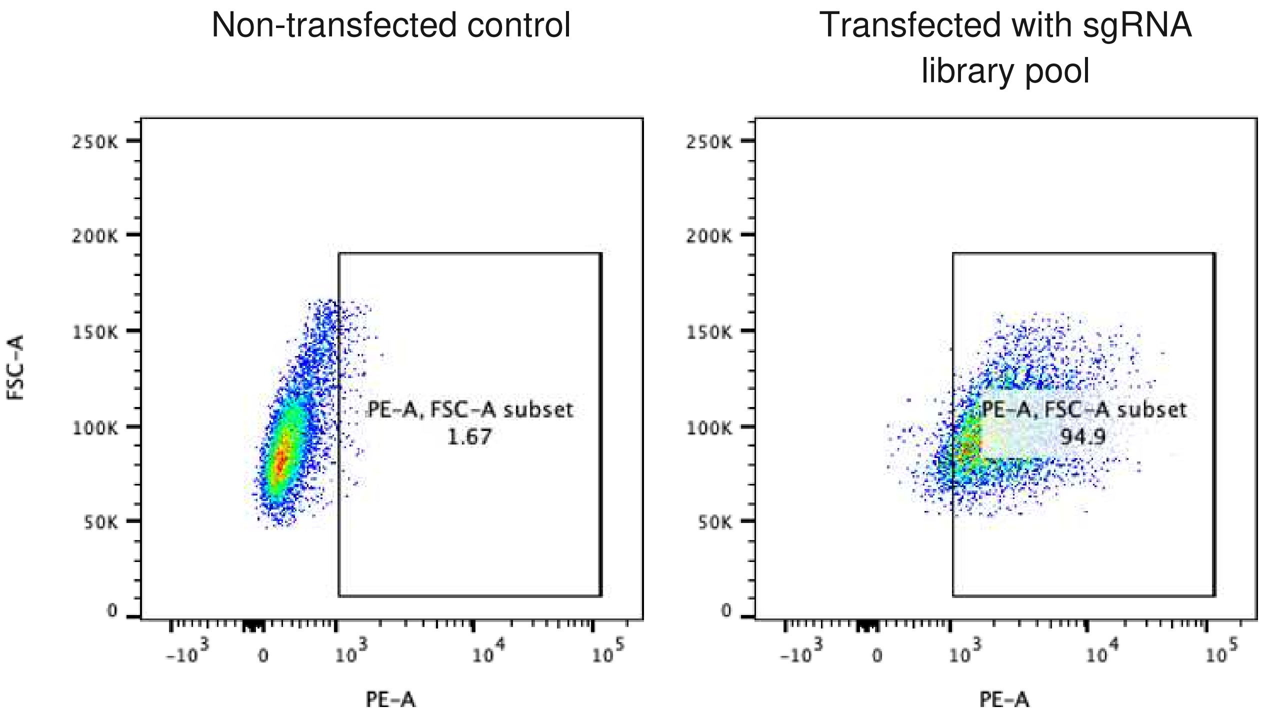

Figure S6: RFP FACS for the perturb-seq assay showing the enrichment of cells transfected with A549-dCas9-KRAB-MeCP at day 14 after puromycin selection. 95% of the cells were RFP positive (PE-A) cells in the sample transfected with the sgRNA library pool, compared to 2% in the non-transfected control sample.

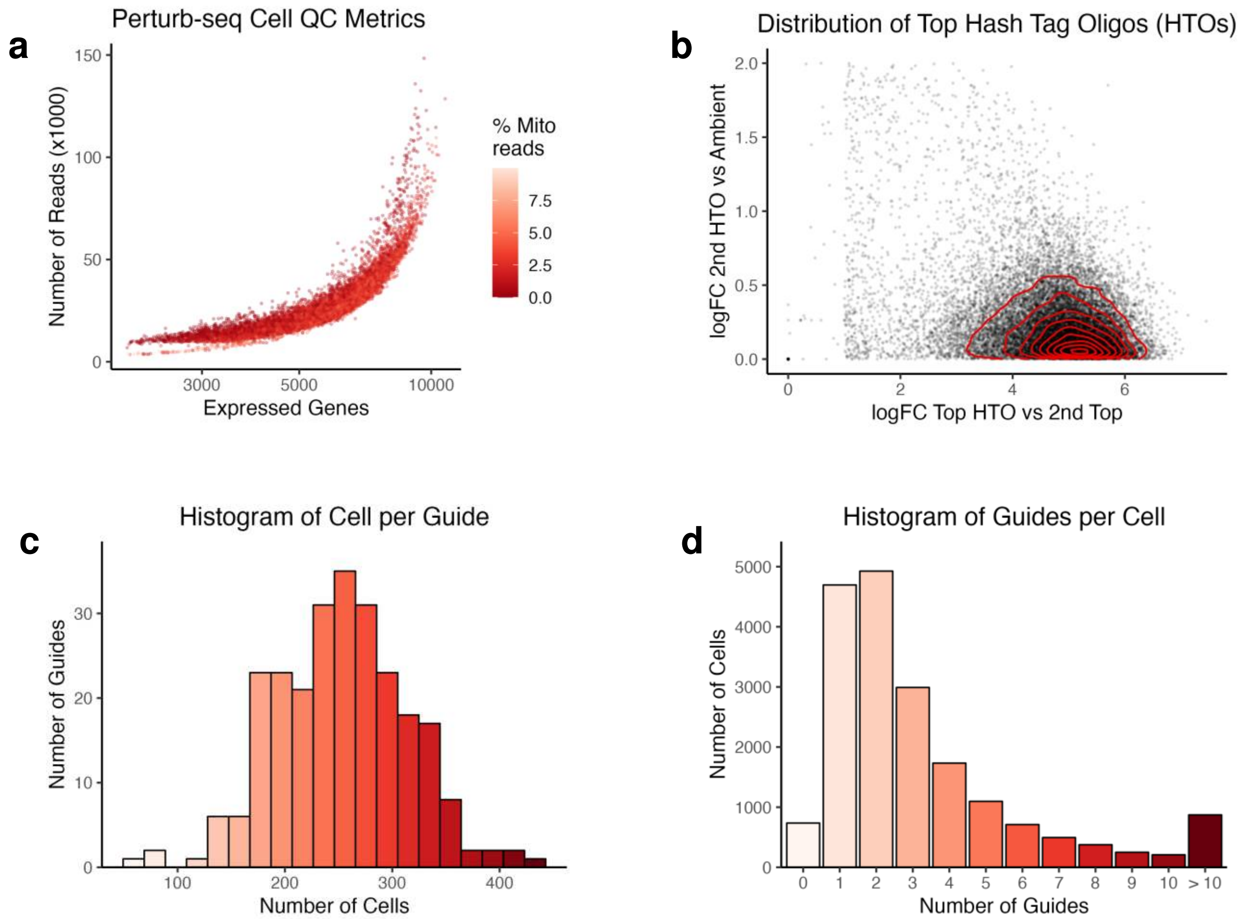

Figure S7: **a**) Scatter plot of quality control metrics of the filtered cells. **b**) Distribution of log2 fold change of the two most abundant oligo-tagged antibodies detected in each cell. **c**) Distribution of the number of cells per guide. **d**) Distribution of the number of guides per cell

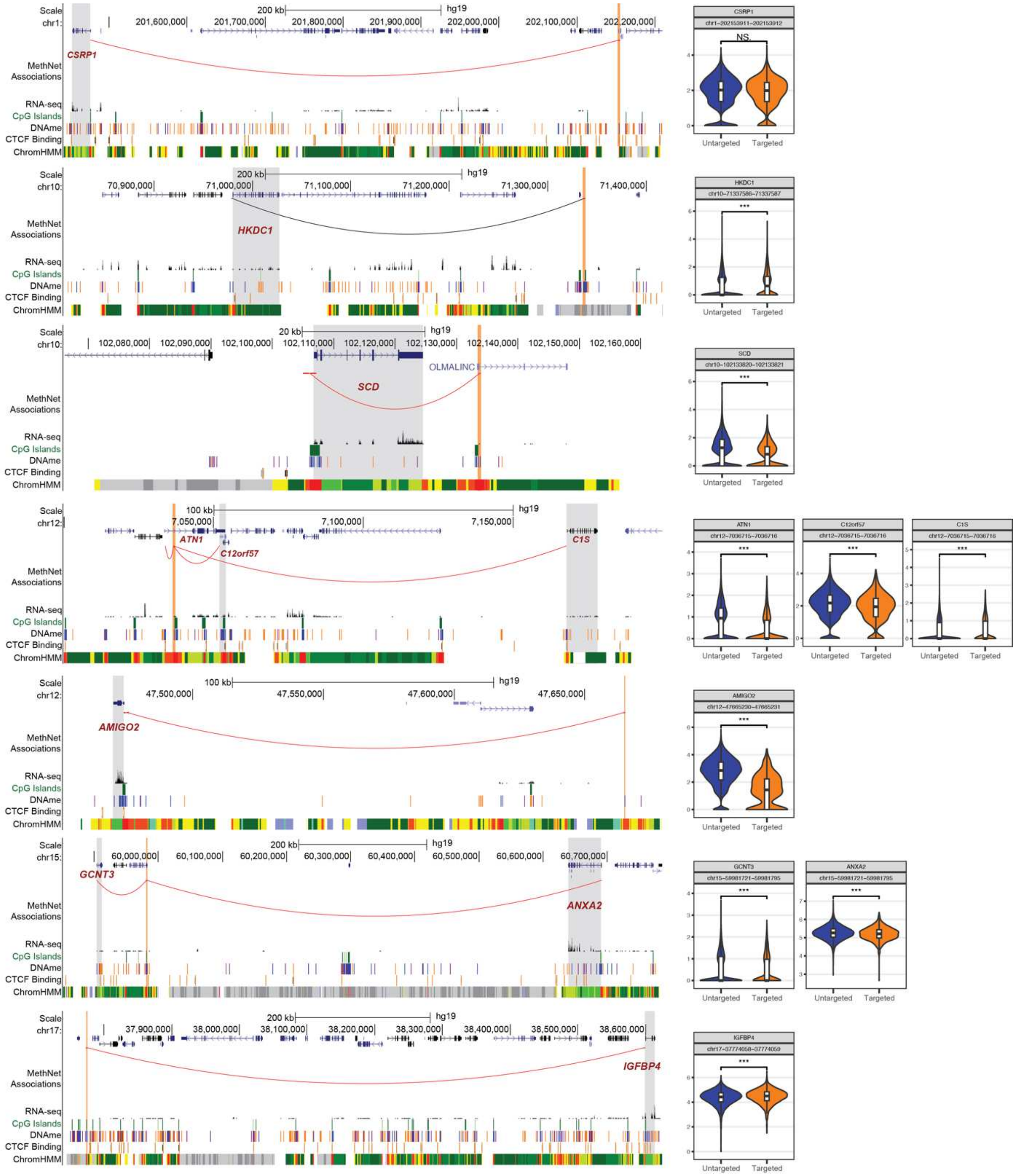

Figure S8. Validation by perturb-seq. Each panel shows the impact of targeting a regulatory region to the corresponding genes. Cells are grouped based on the detection of any sgRNA guide targeting the region.
